# Economic crisis and stillbirth ratios: Evidence from Southern Europe

**DOI:** 10.1371/journal.pone.0259623

**Published:** 2021-11-18

**Authors:** Cleon Tsimbos, Georgia Verropoulou, Dimitra Petropoulou

**Affiliations:** 1 Department of Statistics and Insurance Science, University of Piraeus, Pireas, Greece; 2 Department of Economics, The London School of Economics and Political Science, London, England; University of North Texas Health Science Center, UNITED STATES

## Abstract

In this paper we assess the impact of the recent European recession on stillbirth indices over the course of the 2000s and 2010s; the analysis focuses on four Southern European countries (Greece, Italy, Spain, Portugal), which were seriously affected by the sovereign debt crisis from around 2008 to 2017. We use national vital statistics and established economic indicators for the period 2000–2017; stillbirth ratios (stillbirths per 1000 livebirths) are the chosen response variable. For the purpose of the study, we employ correlation analysis and fit regression models. The overall impact of economic indicators on the stillbirth indices is sizeable and statistically robust. We find that a healthy economy is associated with low and declining levels of stillbirth measures. In contrast, economic recession appears to have an adverse effect (Greece, Italy and Spain), or an unclear impact (Portugal), on the stillbirth outcome. This study provides evidence of the adverse effect of the European sovereign debt crisis and ensuing period of austerity on a scarcely explored aspect of health.

## Introduction

Late fetal deaths are referred to as stillbirths. However, due to differences in definitions and reporting practices, data on stillbirths are usually of low quality. To achieve international statistical comparability the World Health Organization (WHO) recommends defining stillbirths as babies born with no sign of life at or after 28 weeks of gestation.

In 2015, about 2.62 million stillbirths were recorded globally, yet large numbers of stillbirths are preventable through improvements of existing health care systems. Although numbers of stillbirths have been declining in most EU countries and in the US, fetal mortality and preterm births still constitute an important yet often disregarded public health problem. Stillbirth outcome is psychologically burdensome for parents, and results in financial cost for both the family and the state [1–6].

According to official vital registration data (Eurostat database, updated 24.02.2020) circa 2015 stillbirth rates in Europe range from 2.07 (Finland) to 9.40 (France) per 1000 births. The causal links between stillbirth outcome and assorted risk and genetic factors remain largely uncertain despite extensive research, though underlying pathways for stillbirth outcome include adverse placental function (fetal growth retardation, preterm labour), which is largely related to endocrine disruptors. Numerous studies confirm that mothers of low socioeconomic status and unemployed parents face a far higher risk of having a stillbirth or a low birthweight child [7–10]. It has been demonstrated that women or families living in adverse socioeconomic conditions have twice the risk of having a stillborn baby compared to their more privileged counterparts [11]. Unemployment and unfavourable economic conditions are usually associated with poor maternal nutrition, elevated psychological stress during pregnancy and limited access to medical supervision and prenatal care. Several studies show that pregnant women with elevated levels of stress have considerably higher risks of a stillbirth, independent of age, parity, education, smoking and alcohol habits or other compromising characteristics. This is plausible because maternal stress tends to release bioactive mediators and hormones that in turn can stimulate latent pathways, including, among others, neuroendocrine sites, affecting spontaneous preterm deliveries and unfavourable foetal conditions [12–14]. Moreover, in low income countries or deprived environments, poor maternal nutrition and low body mass index can result in higher stillbirth frequency [15].

These findings indicate that fetal mortality is a multifaceted phenomenon encompassing, among others, important socioeconomic aspects. Hence, it is plausible that the deep economic recession experienced by some European countries, may have had an impact on stillbirth rate trends as a result of prevailing stressful economic conditions for individuals and families, as well as through potential deterioration of public health care systems arising from a sustained period of austerity.

The European sovereign debt crisis is the period from 2009 when several countries found themselves unable to meet their public debt obligations, and experienced the collapse of financial institutions, which necessitated a series of government bail-out and bank recapitalisation packages from third-party financial institutions. The key countries experiencing a sovereign debt crisis were Greece, Portugal, Spain and Italy [16]. In addition, Cyprus and Ireland suffered a debt crisis but a little later (in 2012 in Cyprus) or, for a rather short period of time (Ireland). Moreover, affected countries were required to adopt austerity measures to contain public-sector debt as part of the loan agreements, which led to a protracted economic recession and adverse social effects lasting till around 2017.

Amidst rising unemployment and declining incomes, health and social welfare systems struggled to respond [17–19]. Some studies describe the Greek experience in terms of changes in perinatal factors, public hospital attendance, access to healthcare and general mortality during the crisis [20–23]. Others explore the association between the crisis and suicide rates in Greece [24,25], Spain [26,27] and Portugal [28]. Ours is the first contribution to examine the effects on stillbirth indices.

## Aim of the paper

In this paper we use official vital statistics on livebirths and stillbirths and two well established economic indicators for four Southern European countries (Greece, Italy, Spain and Portugal) to investigate the impact of the protracted economic recession on stillbirth indices in the course of the 2000s and 2010s. These countries represent a fairly homogeneous group as they exhibit similar cultural and socioeconomic traits, they share comparable health care systems and welfare regimes, whilst also being the most affected countries of the European sovereign debt crisis since around 2009. Quantitative studies on the relationship between economic conditions and stillbirth indices in the context of an economic recession are very scarce and the present analysis aspires to fill in the gap.

## Data and methods

### Dependent variable

To measure stillbirth levels, two types of indices are used in demographic analyses: the stillbirth ratio, which expresses the number of stillbirths per 1000 livebirths, and the stillbirth rate, which is defined as the ratio of stillbirths per 1000 total births (i.e. stillbirths and livebirths).

The stillbirth rate relates the fetal outcome more closely to the population at risk, so it has a theoretical advantage; however, the measure based on the ratio of stillbirths per 1000 livebirths is considered preferable for international comparisons, due to cross-countries differences in the quality of vital statistics [29–33]. Nevertheless, in low mortality countries, the difference between these measures is trivial in practice, a fact supported by our data. More specifically, preliminary analysis (not shown here) indicated that statistics on stillbirth rates and ratios are very close, time trends are identical whereas application of the regression models using either index as dependent variables provided virtually identical estimates. Hence, for the purpose of the present analysis we use the notion of the stillbirth ratio (*SBR*) as more appropriate:

$$SBR=\frac{Stillbirths}{Livebirths}*1000$$ (1)

In this study we define stillbirths as babies born with no sign of life at or after 28 weeks of gestation (WHO). We consider official national vital registration statistics of four Southern European countries (Greece, Italy, Spain, and Portugal), obtained from the Eurostat data base and selected UN Demographic Yearbooks. The analysis focuses on the period 2000–2017, contrasting the pre-crisis period (2000–2008) and crisis period (2009–2017).

### Independent variables

There are various channels through which the state of the economy can affect policy outcomes, household incomes and well-being and, through these, stillbirth rates; hence, one might consider various macroeconomic indicators. For the purpose of this study we employ alternatively two well-established per capita measures, the Gross National Income (GNI) and the Gross Disposable Income (GDI). GNI is expressed at 2010 prices, while both measures are purchasing power parity adjusted (PPP) to allow for cross-country comparability.

The GNI measures the market value of goods and services produced by all citizens of a country, both domestically and abroad; this index is a core indicator of living standards of nationals. However, given our focus on stillbirth rates, a direct household measure of living standards like GDI is likely to be particularly suitable, as it tracks how the spending power of households evolves. In contrast to GNI, the GDI also accounts for net interest and dividends received and the payment of taxes and social contributions. Since the period of austerity under analysis was characterized by steep rises in taxation across the affected countries, accounting for this is important.

The statistical information on GNI and GDI has been obtained from the World Bank account database (World Development Indicators, Last update 24/4/2019) and the Eurostat data base (Last update 17/5/2019), respectively.

Note that a growing literature questions the representativeness of GNI and GDI as measures of living standards. A particular concern is the trend of growing within-country income inequality which, along with other factors [34–36], implies growth per capita is unequally shared. It is well-documented that median per capita household income has lagged behind the growth in per capita national account measures across OECD countries, reinforcing concerns about over-reliance on the latter as measures of economic well-being. Median income thus emerges as an important additional focal measure [37].

Since the impact of financial crisis may fall disproportionately on lower income households, the repercussions may affect individuals in lower income classes with higher severity. In light of this, we also use median equivalised net household income as an alternative measure of living standards and contrast results with those found using GNI and GDP per capita measures.

### Statistical analysis

To answer our research question two complementary approaches have been implemented.

First, to portray stillbirth ratios trends and their associations with the prevailing national economic conditions, we use graphical presentations (line charts and scatter plots) and linear correlation analysis; patterns as well as the direction of the associations, the intensity and the statistical significance of correlation coefficients before and after the crisis are assessed. This part of the analysis comprises an exploratory display of the data.

Second, we fit OLS regression models to examine the hypothesis that the relationship between economic indicators and stillbirth ratios differentiates before and during the crisis. To evaluate the change in the above-mentioned relationships between the two periods under investigation (2000–2008, 2009–2017) we employ a dummy variable approach [38].

Let:

- *Y* be the dependent variable under research, i.e. the stillbirth ratio of the population (number of stillbirths per 1000 livebirths)
- *D* a dummy variable taking the values of 0 for the period 2000–2008, and 1 for the period 2009–2017
- *X* the explanatory variable referring to either the GNI or the GDI (both per capita and PPP-adjusted) which are considered to reflect the national and household economic conditions of a country.

Pooling the year-by-year observations we consider the regression model:

$$Y_{t}=b_{0}+b_{1}\cdot D_{t}+b_{2}\cdot X_{t}+b_{3}\cdot \left(D_{t}\cdot X_{t}\right)+u_{t}$$ (2)

Applying ordinary least squares (OLS) the estimated model is:

$${\hat{Y}}_{t}={\hat{b}}_{0}+{\hat{b}}_{1}\cdot D_{t}+{\hat{b}}_{2}\cdot X_{t}+{\hat{b}}_{3}\cdot \left(D_{t}\cdot X_{t}\right)$$ (3)

The regression coefficient *b*_1_ represents the *differential intercept* while *b*_3_ is the *differential slope* pointing out how much the slope of the estimated quantitative relationship of the first period (2000–2008) differs from the slope of the second period (2009–2017).

Assuming that all OLS properties are fulfilled we obtain the following models:

$$E\left(Y_{t}\;|\;D_{t}=0,\;X_{t}\right)=b_{0}+b_{2}\cdot X_{t}$$ (4)

$$E\left(Y_{t}\;|\;D_{t}=1,\;X_{t}\right)=\left(b_{0}+b_{1}\right)+\left(b_{2}+b_{3}\right)\cdot X_{t}$$ (5)

which express the conditional mean stillbirth ratios of the sub-periods 2000–2008 (pre-crisis) and 2009–2017 (crisis), respectively.

The regression models are estimated by country, introducing alternatively the indicators GDI and DNI as independent variables to test our hypothesis more comprehensively. The OLS assumptions are assessed employing established tests. The autocorrelation assumption is evaluated using the Durbin-Watson test (D-W), the normality assumption of the error term is assessed on the basis of the Smirnov-Kolmogorov test of normality (S-K) and the heteroscedasticity assumption on the basis of the Breusch-Pagan-Golfrey test III (B-P-G). The statistical analysis was carried out using SPSS version 24 and Stata 13.

While the OLS regression does not account for confounders affecting both the dependent and the independent variables (for instance, poverty levels) as would an instrumental variable approach, use of the latter technique is precluded by our small sample size, which can result in biased findings [39,40].

To assess the robustness of the OLS regression findings a panel fixed effects regression approach is employed. The panel regression accounts for individual heterogeneity (i.e. differences at country level), while fixed effects are preferable when analysing the impact of time-varying variables [41].

Finally, as a further robustness check we contrast the trends of per capital GDI with those of the median equivalised net income (hereafter median income) while we also re-run our regression analysis using median income as independent variable. Median equivalised net, or disposable, income, is the median of total income of all households, after tax and other deductions, that is available for spending or saving, divided by the number of household members converted into equivalised adults; household members are equalised or made equivalent by weighting each according to their age, using the so-called modified OECD equivalence scale. The data used in the analysis were obtained from Eurostat (last update of the data 1.7.2021; extracted on19.7.2021: https://measuring-progress.eu/median-equivalised-net-income). It should be noted that the relevant values for Greece for 2002 and for Spain, Italy and Portugal for 2002–2003 are estimated using linear interpolation between adjacent years, as they were missing.

## Results

### Descriptive findings

**Fig 1:**
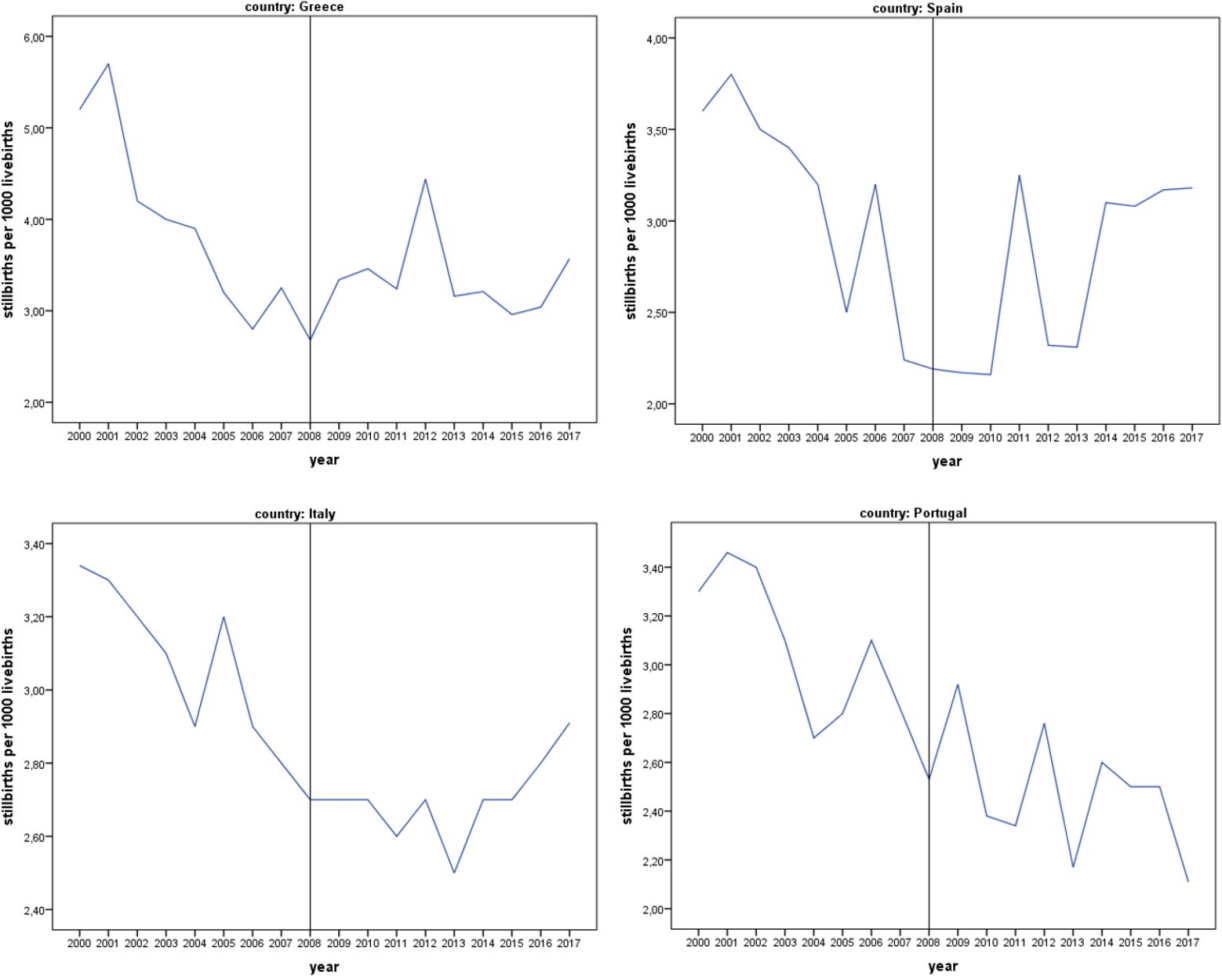
Trends in stillbirth ratios by country: 2000–2017.

During the period 2000–2008 a rather clear downward trend in the stillbirth ratios is observed in all countries under investigation, though the ratios for Greece stand at a slightly higher level throughout the period, compared to all other countries. In contrast, during the period 2009–2017 the annual series of the stillbirth indicators exhibit an unclear pattern, characterized by either strong fluctuations (Spain, Portugal) or temporal increases (Italy, Greece) particularly in 2016 and 2017 (Fig 1).

**Fig 2:**
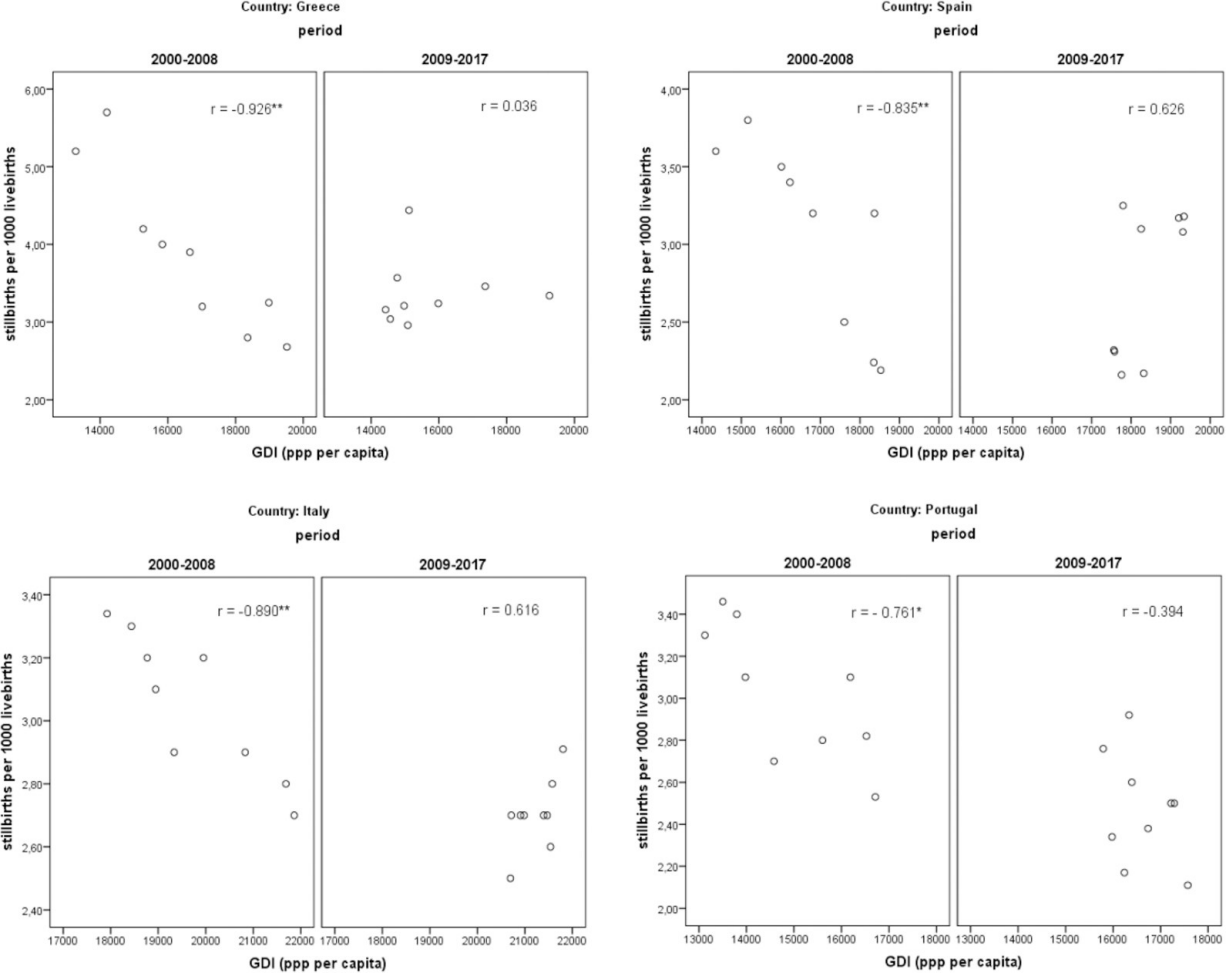
Scatter plots and correlation coefficients between stillbirth ratios and Gross Disposable Income (GDI). (** statistically significant at 0.01 * statistically significant at 0.05).

**Fig 3:**
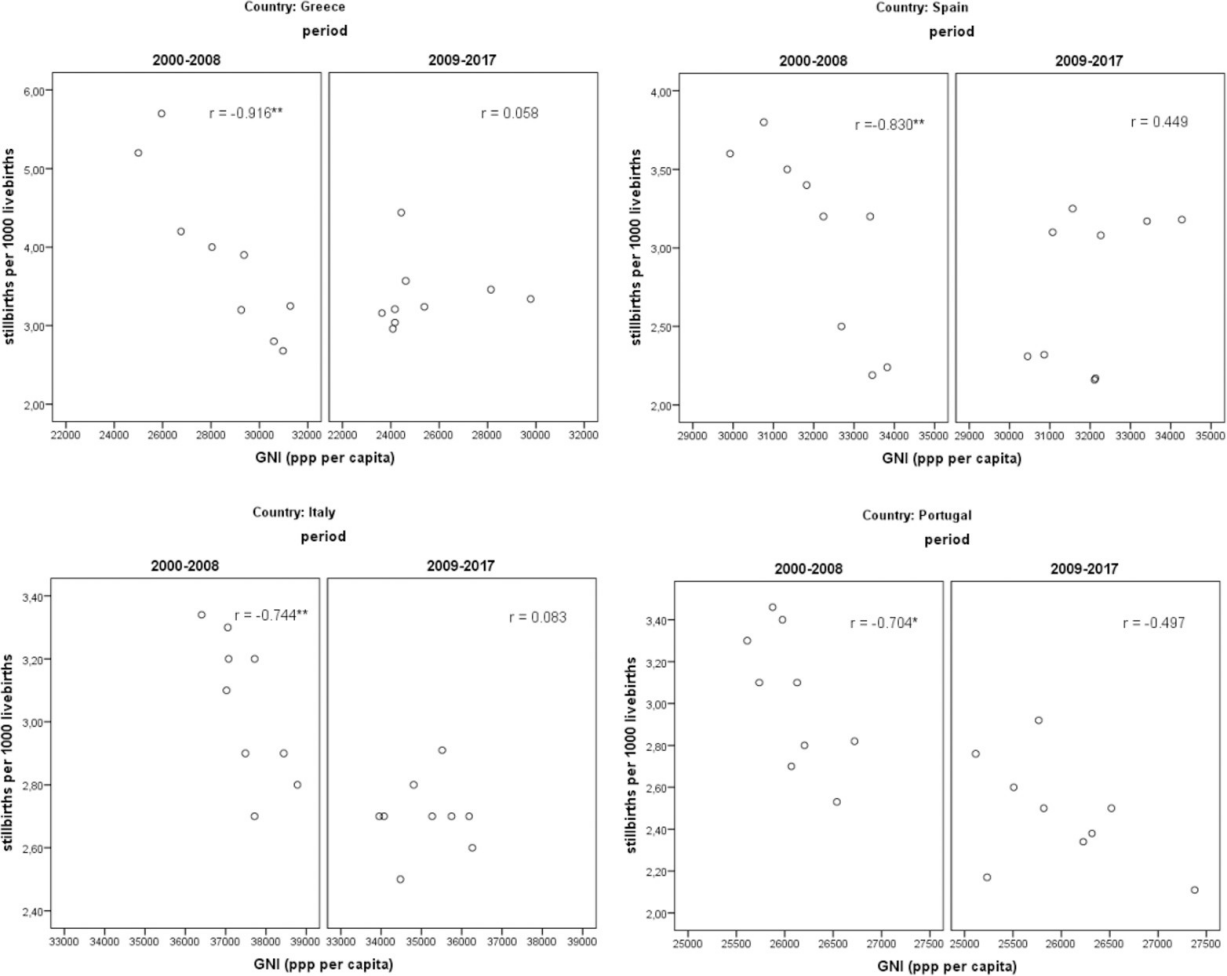
Scatter plots and correlation coefficients between stillbirth ratios and Gross National Income (GNI). (** statistically significant at 0.01 * statistically significant at 0.05).

The pattern becomes more apparent when examining the relationship between stillbirth ratios and economic conditions in the two periods (Figs 2 and 3). In all countries, during the pre-crisis period, the correlation coefficients between stillbirth ratios and both economic measures are negative, high in value and statistically significant, implying that good socioeconomic conditions act favorably and contribute towards low and declining stillbirth outcomes over time. The correlation coefficients between stillbirth ratios and GDI range from -0.761 (Portugal) to -0.926 (Greece) and between stillbirth ratios and GNI range from -0.704 (Portugal) to -0.966 (Greece). In contrast, during the period of adversity (2009–2017) the correlation coefficients become positive but are not statistically significant, with the exception of Portugal where the coefficient remains negative but becomes statistically insignificant and small in magnitude.

These findings provide a first indication that the relationship between prevailing economic conditions and stillbirth ratios differentiates between the two sub-periods under study in all countries. In the pre-crisis period, the estimated relationships are clearly negative, as expected, while in the post-crisis period the relationships are indistinct.

### Regression results

Between 2000 and 2008 GDI increased in all countries (not shown here) and more markedly in Greece (47%). Thereafter, declines were recorded, minor ones for Portugal and Spain, followed by recovery and return to the pre-crisis levels in 2015, a slight but more protracted one for Italy and a sharp decrease for Greece (23%) where recovery was modest. GNI showed only slight fluctuations in Portugal in the overall period. In Spain, Italy and Greece it followed roughly the trends described for the GDI. The index in Spain had recovered by 2016 but not in the other two countries.

Tables 1 and 2 present the effect of GDI and GNI on the stillbirth ratios based on the regression models. All estimated models satisfy the OLS assumptions (criteria: D-W, S-K, B-P-G) and have a good fit (based on the R^2^ and the F-test). From a statistical point of view all models work well; however, the fit is slightly better when GDI is used as independent variable, perhaps because it expresses individual wellbeing more accurately, while also exhibiting more substantial variation.

**Table 1:** Regression results by country: Explanatory variable is Gross Disposable Income (GDI) per capita PPP-adjusted.

		b	SE	t	Sig.
Greece (N = 18)	(Constant)	11.334	1.233	9.193	.000
	D	-8.111	1.985	-4.087	.001
	GDI	-.450	.074	-6.090	.000
	D^**.**^ GDI	.460	.123	3.736	.002
	R^2^ = 0.868	F = 14.253 (*p* = 0.000)	D-W d = 2.309 d_L_ = 0.933 d_U_ = 1.696	S-K = 0.193 (*p* = 0.075)	B-P-G = 0.954 (χ^2^ = 7.815)
Spain (N = 18)	(Constant)	8.720	1.517	5.749	.000
	D	-13.442	3.639	-3.694	.002
	GDI	-.336	.090	-3.738	.002
	D^**.**^ GDI	.743	.201	3.691	.002
	R^2^ = 0.783	F = 7.418 (*p* = 0.003)	D-W d = 2.303 d_L_ = 0.933 d_U_ = 1.696	S-K = 0.143 (*p* = 0.200)	B-P-G = 3,096 (χ^2^ = 7.815)
Italy (N = 18)	(Constant)	5.887	.512	11.497	.000
	D	-6.819	1.975	-3.452	.004
	GDI	-.144	.026	-5.556	.000
	D^**.**^ GDI	.315	.093	3.368	.005
	R^2^ = 0.926	F = 28.157 (*p* = 0.000)	D-W d = 2.445 d_L_ = 0.933 d_U_ = 1.696	S-K = 0.138 (*p* = 0.200)	B-P-G = 0.378 (χ^2^ = 7.815)
Portugal (N = 18)	(Constant)	6.307	.719	8.775	.000
	D	-1.450	5.511	-.263	.796
	GDI	-.222	.046	-4.820	.000
	D^**.**^ GDI	.079	.339	.233	.819
	R^2^ = 0.828	F = 10.170 (*p* = 0.001)	D-W d = 2.091 d_L_ = 0.933 d_U_ = 1.696	S-K = 0.107 (*p* = 0.200)	B-P-G = 3.924 (χ^2^ = 7.815)

Note:

D-W d: Durbin-Watson d statistic for serial correlation (d_L_ and d_U_ are the Lower and Upper bounds respectively of the estimated d).

S-K: Smirnov-Kolmogorov test of normality and corresponding significance level (*p*).

B-P-G: Breusch-Pagan-Golfrey test III statistic for heteroscedasticity and corresponding chi-squared (χ^2^) value.

**Table 2:** Regression results by country: Explanatory variable is Gross National Income (GNI) per capita PPP-adjusted.

		b	SE	t	Sig.
Greece (N = 18)	(Constant)	15.824	2.036	7.771	.000
	D	-12.752	2.811	-4.536	.000
	GNI	-.418	.071	-5.882	.000
	D^**.**^ GNI	.430	.104	4.129	.001
	R^2^ = 0.741	F = 13.337 (*p* = 0.000)	D-W d = 2.585 d_L_ = 0.933 d_U_ = 1.696	S-K = 0.202 (*p* = 0.070)	B-P-G = 1.098 (χ^2^ = 7.815)
Spain (N = 18)	(Constant)	15.283	3.586	4.262	.001
	D	-18.248	5.261	-3.469	.004
	GNI	-.380	.111	-3.409	.004
	D^**.**^ GNI	.558	.164	3.407	.004
	R^2^ = 0.511	F = 5.498 (*p* = 0.010)	D-W d = 1.987 d_L_ = 0.933 d_U_ = 1.696	S-K = 0.123 (*p* = 0.200)	B-P-G = 2.682 (χ^2^ = 7.815)
Italy (N = 18)	(Constant)	11.686	2.576	4.536	.000
	D	-9.371	3.310	-2.831	.013
	GNI	-.230	.069	-3.353	.005
	D^**.**^ GNI	.241	.091	2.662	.019
	R^2^ = 0.727	F = 12.442 (*p* = 0.000)	D-W d = 1.751 d_L_ = 0.933 d_U_ = 1.696	S-K = 0.184 (*p* = 0.109)	B-P-G = 0.864 (χ^2^ = 7.815)
Portugal (N = 18)	(Constant)	18.782	4.778	3.931	.002
	D	-13.267	8.420	-1.576	.137
	GNI	-.607	.182	-3.333	.005
	D^**.**^ GNI	.491	.326	1.509	.154
	R^2^ = 0.535	F = 5.363 (*p* = 0.011)	D-W d = 1.846 d_L_ = 0.933 d_U_ = 1.696	S-K = 0.117 (*p* = 0.200)	B-P-G = 2.160 (χ^2^ = 7.815)

Note:

D-W d: Durbin-Watson d statistic for serial correlation (d_L_ and d_U_ are the Lower and Upper bounds respectively of the estimated d).

S-K: Smirnov-Kolmogorov test of normality and corresponding significance level (*p*).

B-P-G: Breusch-Pagan-Golfrey test III statistic for heteroscedasticity and corresponding chi-squared (χ^2^) value.

With respect to Greece, Spain and Italy, all regression coefficients of GDI (and GNI) have, as expected, a negative sign and are statistically significant, reflecting the inverse association between stillbirth ratios and economic conditions. As the differential intercepts and the differential slopes are statistically significant, the estimated models point out that the association between the independent and the dependent variable in the two sub-periods (2000–2008, 2009–2017) differentiates; this finding holds using either the GDI or the GNI as independent variable.

For Portugal, on the other hand, the results show that good economic conditions have a favorable effect on the stillbirth ratios, but the coefficients of the differential intercepts and differential slopes, though they have the expected sign, are not statistically significant, indicating that the difference in the quantitative relationships for the two sub-periods 2000–2008 and 2009–2017 is negligible.

### Robustness checks

The findings of the panel regression fixed effects analysis, presented in Table 3, are consistent with the OLS results for both the GDI and the GNI, indicating that our conclusions based on the OLS are robust. Controlling for heterogeneity between countries, both the GDI and the GNI exhibit a statistically significant inverse association with stillbirth ratios, which differentiates between the two periods under investigation.

**Table 3:** Robustness check: Panel regression results.

Explanatory variable		b	SE	t	Sig.
GDI	(Constant)	7.447	.642	11.60	.000
	D	-3.049	.744	-4.10	.000
	GDI	-.246	.037	-6.57	.000
	D^**.**^ GDI	.159	.042	3.79	.001
	rho = .5924				
GNI	(Constant)				
	D	-2.187	.745	-2.94	.005
	GNI	-.166	.037	-4.44	.000
	D GNI	.051	.024	2.10	.040
	rho = .6908				

**Fig 4:**
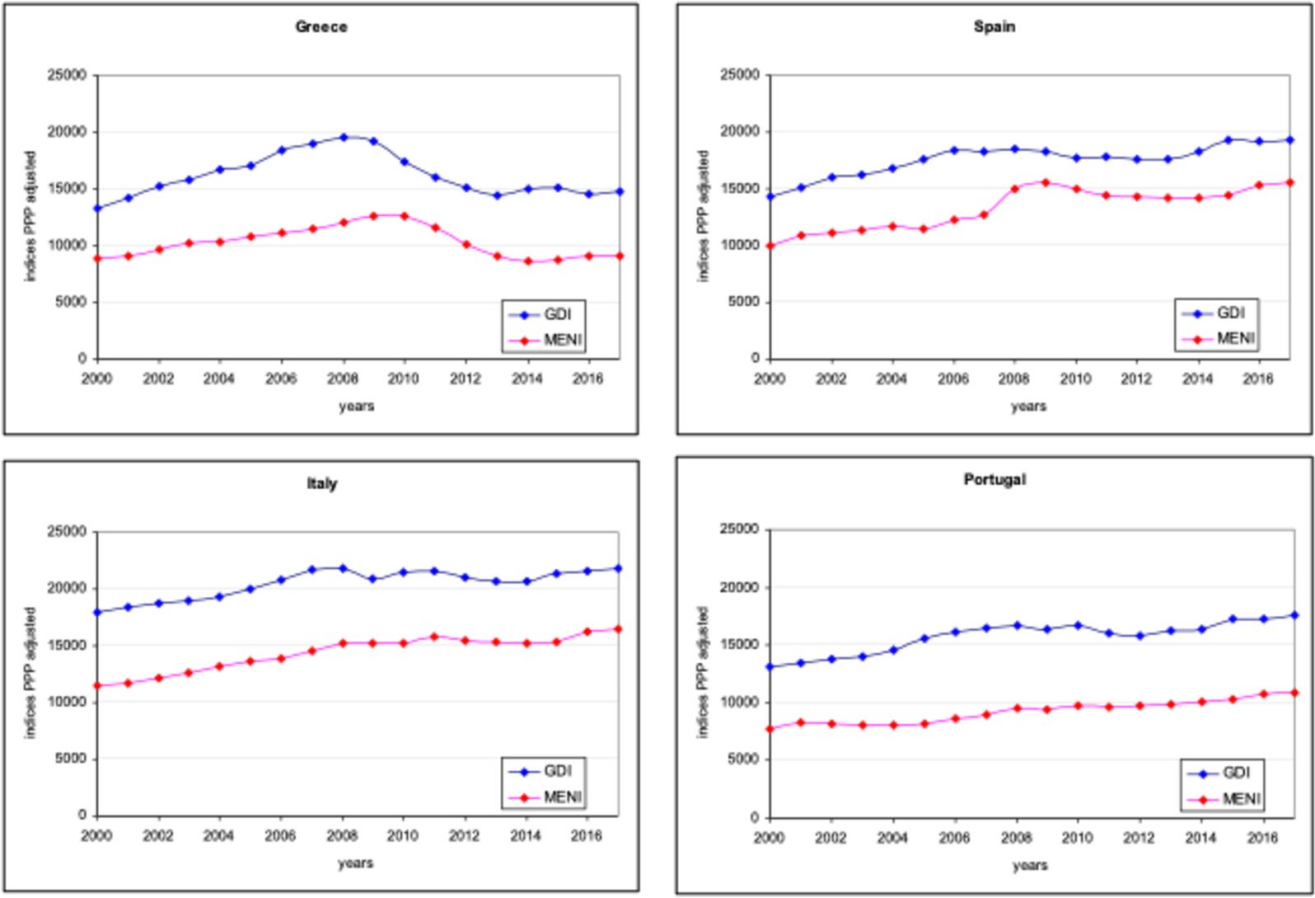
Time trends of Gross Disposable Income per capita PPP adjusted (GDI) and Equivalised Median Net Income PPP adjusted (MENI) by country: 2000–2017.

Contrasting the trends of per capital GDI and median income, the findings indicate that these measures are strongly and statistically significantly correlated (Appendix, Table 4 and Fig 4); this also holds regarding GNI and median income (results not shown here). Re-running our regression analysis using median income as the independent variable indicates that our results are generally robust to the use of median income (Appendix, Table 5), except for Spain, where a statistically significant relationship between median income and stillbirth rates is not found.

**Table 4:** Correlation coefficients between Equivalised Median Net Income PPP adjusted (MENI) and Gross Disposable Income per capita PPP adjusted (GDI) by country (2000–2017).

Correlations				
country			MENI	GDI
Greece	MENI	Pearson Correlation	1	,873**
		Sig. (2-tailed)		,000
		N	18	18
	GDI	Pearson Correlation	,873**	1
		Sig. (2-tailed)	,000	
		N	18	18
Spain	MENI	Pearson Correlation	1	,826**
		Sig. (2-tailed)		,000
		N	18	18
	GDI	Pearson Correlation	,826**	1
		Sig. (2-tailed)	,000	
		N	18	18
Italy	MENI	Pearson Correlation	1	,935**
		Sig. (2-tailed)		,000
		N	18	18
	GDI	Pearson Correlation	,935**	1
		Sig. (2-tailed)	,000	
		N	18	18
Portugal	MENI	Pearson Correlation	1	,863**
		Sig. (2-tailed)		,000
		N	18	18
	GDI	Pearson Correlation	,863**	1
		Sig. (2-tailed)	,000	
		N	18	18

**. Correlation is significant at the 0.01 level (2-tailed).

**Table 5:** Regression results by country: Explanatory variable is Median Equivalised Net Income (MENI) PPP-adjusted.

		b	SE	t	Sig.
Greece (N = 18)	(Constant)	13.279	1.452	9.142	.000
	D	-10.371	1.726	-6.010	.000
	MENI	-.905	.139	-6.501	.000
	D^**.**^ MENI	.951	.166	5.729	.000
	R^2^ = 0.777	F = 16.265 (*p* = 0.000)	D-W d = 2.373 d_L_ = 0.933 d_U_ = 1.696	S-K = 0.244 (*p* = 0.006)	B-P-G = 0.378 (χ^2^ = 7.815)
Spain (N = 18)	(Constant)	6.985	1.369	5.102	.000
	D	-3.993	4.489	-.890	.389
	MENI	-.331	.115	-2.878	.012
	D^**.**^ MENI	.314	.311	1.010	.329
	R^2^ = 0.427	F = 3.475 (*p* = 0.045)	D-W d = 2.042 d_L_ = 0.933 d_U_ = 1.696	S-K = 0.214 (*p* = 0.029)	B-P-G = 2.790 (χ^2^ = 7.815)
Italy (N = 18)	(Constant)	5.209	.361	14.441	.000
	D	-5.057	1.189	-4.253	.001
	MENI	-.164	.027	-6.013	.000
	D^**.**^ MENI	.327	.078	4.222	.001
	R^2^ = 0.875	F = 32.711 (*p* = 0.000)	D-W d = 2.734 d_L_ = 0.933 d_U_ = 1.696	S-K = 0.177 (*p* = 0.139)	B-P-G = 0.504 (χ^2^ = 7.815)
Portugal (N = 18)	(Constant)	6.084	1.527	3.984	.001
	D	-1.429	2.428	-.589	.565
	MENI	-.364	.180	-2.021	.050
	D^**.**^ MENI	.147	.261	.564	.581
	R^2^ = 0.631	F = 7.973 (*p* = 0.002)	D-W d = 1.975 d_L_ = 0.933 d_U_ = 1.696	S-K = 0.106 (*p* = 0.200)	B-P-G = 2.880 (χ^2^ = 7.815)

Note:

D-W d: Durbin-Watson d statistic for serial correlation (d_L_ and d_U_ are the Lower and Upper bounds respectively of the estimated d).

S-K: Smirnov-Kolmogorov test of normality and corresponding significance level (*p*).

B-P-G: Breusch-Pagan-Golfrey test III statistic for heteroscedasticity and corresponding chi-squared (χ^2^) value.

## Discussion and conclusions

This paper augments the existing literature exploring the socioeconomic determinants of stillbirth indices to examine the impact of the state of the economy on stillbirths. The European sovereign debt crisis led to an economic recession that was both deep and protracted, lasting from around 2009 to around 2017, a period long enough for shifts in patterns of stillbirth indices to be detected. Moreover, the fact that several countries were affected presents the opportunity to compare findings from several Southern European countries (Greece, Portugal, Italy and Spain).

For the purpose of the analysis, we estimate regression models using annual data for the period 2000–2017, by country and, to increase the robustness of the results, separately for two indicators, the GDI and the GNI. The fact that the models have a somewhat better fit for GDI may indicate that the ability of individuals to pay for private health care in times of hardship and cutbacks in public health expenditure have a greater effect on stillbirths than average country prosperity. Moreover, with sharp tax hikes over the period in most affected countries, a measure of disposable income is likely to more accurately reflect household ability to make out-of-pocket health expenditures.

Our findings indicate that, overall, there was an undesirable and statistically significant effect of the debt crisis on the stillbirth ratios in Southern Europe. During periods of economic stability and prosperity, economic indicators are associated with low and declining levels of stillbirth ratios. In contrast, since the onset of the crisis, an unfavorable effect on the stillbirth indices is confirmed for Greece, Italy and Spain.

With respect to Portugal, the associations between economy and stillbirth ratios in the long run is statistically significant and in the expected direction, but the quantitative relationships do not differ in the pre- and post-crisis periods. Perhaps because the Portuguese crisis was less severe than elsewhere and of shorter duration, with Portugal emerging from the crisis earlier in 2014. In fact, the GNI showed little change in the period 2008–2017, whereas the GDI showed the least variation for Portugal amongst the countries considered. It is also worth noting that further analysis (not presented here) considering countries only marginally affected by the crisis (e.g. Austria, Belgium, the Netherlands, Denmark, Finland etc.) showed virtually no effect of the crisis on stillbirth ratios, while robustness checks reinforce the validity of our findings concerning Southern European countries.

Other factors that may account for the above-mentioned disparities between Southern European countries may relate to differences in the health system. For example, Greece has an employer-based health insurance system with multiple social insurance funds. As crisis affects employment, so too does it affect access to social insurance. In contrast pre-natal healthcare in Portugal is free and not linked to a social insurance fund, which might make stillbirth indices more resilient in Portugal during crisis. More broadly, Portuguese per capital household out of pocket health expenditure is systematically lower than that of the other three countries (as measured in current US$) throughout the period of analysis (WHO Global Health Expenditure database), suggesting a potentially weaker relationship between GDI and health outcomes.

Economic crisis tends to affect harder the most disadvantaged segments of the population, while it may result in a decrease in the availability and quality of public health care. These may have undesirable consequences at the community level and increase socioeconomic inequalities in health outcomes, since personal affluence may play a significant role. Hence, the implication of the present analysis is that in times of crisis maternal and child health should comprise a priority for policy makers.
